# Supplementary material for: Robust priors for regularized regression
Source: Cogn Psychol. 2022 Feb;132:None. doi: 10.1016/j.cogpsych.2021.101444 (PMC8903146; doi:10.1016/j.cogpsych.2021.101444)
Supplement: MMC S1 — See SI for optimal weight derivations, model validation, penalty parameter list and alternative training scheme. [file mmc1.pdf]

Supplementary Information
Robust priors for regularized regression

Sebastian Bobadilla-Suarez,[1*] Matt Jones,[2] Bradley C. Love[1,3]

[1]Department of Experimental Psychology, University College London
26 Bedford Way, London, WC1H 0AP, UK
[2]Psychology and Neuroscience, University of Colorado Boulder
Boulder, CO 80309-0345, USA
[3]The Alan Turing Institute
96 Euston Road, London, NW1 2DB, UK

[*]To whom correspondence should be addressed; E-mail: sebastian.suarez.12@ucl.ac.uk.

Derivation of $\hat{\boldsymbol{w}}_{ridge}$

The following is a derivation of the weights for the general penalized regression model defined in Equation 1 of the main text. The derivation applies to standard ridge regression (when $\boldsymbol{w}_{prior} = \overrightarrow{0}$) and also to our robust models TAL-prior and TTB-prior when $\boldsymbol{w}_{prior} = \boldsymbol{w}_{TAL\ prior}$ or $\boldsymbol{w}_{prior} = \boldsymbol{w}_{TTB\ prior}$, respectively.

Given a value of $\theta \geq 0$, the weights are defined by

$$\hat{\boldsymbol{w}}_{ridge} = \arg\min_{\boldsymbol{w}} \{\|\boldsymbol{y} - \boldsymbol{X}\boldsymbol{w}\|^2 + \theta \|\boldsymbol{w} - \boldsymbol{w}_{prior}\|_2^2\} \tag{1}$$

$$= \arg\min_{\boldsymbol{w}} \{(\boldsymbol{y} - \boldsymbol{X}\boldsymbol{w})^T(\boldsymbol{y} - \boldsymbol{X}\boldsymbol{w}) + \theta(\boldsymbol{w} - \boldsymbol{w}_{prior})^T(\boldsymbol{w} - \boldsymbol{w}_{prior})\}. \tag{2}$$

This objective function is convex and differentiable, so the minimum can be obtained by differentiating:

$$\overrightarrow{0} = \frac{\partial}{\partial \boldsymbol{w}} \left[(\boldsymbol{y} - \boldsymbol{X}\boldsymbol{w})^T(\boldsymbol{y} - \boldsymbol{X}\boldsymbol{w}) + \theta(\boldsymbol{w} - \boldsymbol{w}_{prior})^T(\boldsymbol{w} - \boldsymbol{w}_{prior})\right]_{\boldsymbol{w}=\hat{\boldsymbol{w}}_{ridge}}$$

$$= -2\boldsymbol{X}^T(\boldsymbol{y} - \boldsymbol{X}\hat{\boldsymbol{w}}_{ridge}) + 2\theta(\hat{\boldsymbol{w}}_{ridge} - \boldsymbol{w}_{prior}), \tag{3}$$

implying

$$\left(\boldsymbol{X}^T\boldsymbol{X} + \theta \boldsymbol{I}_n\right)\hat{\boldsymbol{w}}_{ridge} = \boldsymbol{X}^T\boldsymbol{y} + \theta \boldsymbol{w}_{prior} \tag{4}$$

and therefore

$$\hat{\boldsymbol{w}}_{ridge} = \left(\boldsymbol{X}^T\boldsymbol{X} + \theta \boldsymbol{I}_n\right)^{-1}\left(\boldsymbol{X}^T\boldsymbol{y} + \theta \boldsymbol{w}_{prior}\right) \tag{5}$$

where $\boldsymbol{I}_n$ is the $n \times n$ identity matrix.

Derivation of $\hat{\boldsymbol{w}}_{log(ridge)}$

Here we derive the optimal weights for logistic ridge regression, $\hat{\boldsymbol{w}}_{log(ridge)}$, as defined in Equation 15 of the main text. We expand on the derivation in van Wieringen (2015) to incorporate our prior weight vector $\boldsymbol{w}_{prior}$ in the penalty term, $\frac{1}{2}\theta\|\boldsymbol{w}-\boldsymbol{w}_{prior}\|_2^2$. We approximate the optimal solution with the iterative Newton-Raphson algorithm, using the superscript t to index iterations. Thus, $\hat{\boldsymbol{w}}^0$ is an initial vector for the first iteration (e.g., $\hat{\boldsymbol{w}}^0 = \overrightarrow{\mathbf{0}}$). For a complete explanation please see (**?**).

The loss function for penalized logistic regression can be written as

$$\begin{aligned}\mathcal{L}(\boldsymbol{w};\boldsymbol{X},\boldsymbol{y}) &= -\ln \Pr[\boldsymbol{y}|\boldsymbol{X},\boldsymbol{w}] + \tfrac{1}{2}\theta\|\boldsymbol{w}-\boldsymbol{w}_{prior}\|_2^2 \\ &= -\sum_{i=1}^{n}\left(y_i\boldsymbol{X}_{i\cdot}\boldsymbol{w} - \ln\left[1+\exp(\boldsymbol{X}_{i\cdot}\boldsymbol{w})\right]\right) + \tfrac{1}{2}\theta\|\boldsymbol{w}-\boldsymbol{w}_{prior}\|_2^2. \end{aligned} \tag{6}$$

where $\boldsymbol{X}_{i\cdot}$ is the ith row of $\boldsymbol{X}$ and $y_i \in \{0,1\}$ is the ith observation. We minimize this loss by approximating the vector $\hat{\boldsymbol{w}}_{log(ridge)}$ where the gradient is zero, by applying the Newton-Raphson algorithm to $\frac{\partial\mathcal{L}}{\partial\boldsymbol{w}}$. The update step is

$$\boldsymbol{w}^t = \boldsymbol{w}^{t-1} - \left(\frac{\partial^2\mathcal{L}}{\partial\boldsymbol{w}\partial\boldsymbol{w}^T}\right)^{-1}\Bigg|_{\boldsymbol{w}=\boldsymbol{w}^{t-1}} \frac{\partial\mathcal{L}}{\partial\boldsymbol{w}}\Bigg|_{\boldsymbol{w}=\boldsymbol{w}^{t-1}} \tag{7}$$

Thus, we need the gradient and the Hessian of $\mathcal{L}$. The gradient is given by

$$\frac{\partial\mathcal{L}}{\partial\boldsymbol{w}} = \boldsymbol{X}^T\boldsymbol{g} + \theta(\boldsymbol{w}-\boldsymbol{w}_{prior}) \tag{8}$$

where

$$g_i = \frac{\exp(\boldsymbol{X}_{i\cdot}\boldsymbol{w})}{1+\exp(\boldsymbol{X}_{i\cdot}\boldsymbol{w})} - y_i. \tag{9}$$

The Hessian is given by

$$\frac{\partial^2\mathcal{L}}{\partial\boldsymbol{w}\partial\boldsymbol{w}^T} = \boldsymbol{X}^T\boldsymbol{U}\boldsymbol{X} + \theta \tag{10}$$

where $\boldsymbol{U}$ is a diagonal matrix with

$$U_{ii} = \frac{\exp(\boldsymbol{X}_{i\cdot}\boldsymbol{w}^{t-1})}{(1+\exp(\boldsymbol{X}_{i\cdot}\boldsymbol{w}^{t-1}))^2} \tag{11}$$

.

Therefore the Newton-Raphson update for the estimated weights $\hat{\boldsymbol{w}}_{log(ridge)}$ of our penalized logistic regression model is:

$$\hat{\boldsymbol{w}}^{t}_{log(ridge)} = \hat{\boldsymbol{w}}^{t-1}_{log(ridge)} - \boldsymbol{V}^{-1}\left[\boldsymbol{X}^T\boldsymbol{g} + \theta\left(\hat{\boldsymbol{w}}^{t-1}_{log(ridge)} - \boldsymbol{w}_{prior}\right)\right] \tag{12}$$

where $\boldsymbol{V} = \boldsymbol{X}^T\boldsymbol{U}\boldsymbol{X} + \theta\boldsymbol{I}$. We obtain our final estimate $\hat{\boldsymbol{w}}_{log(ridge)}$ when $\hat{\boldsymbol{w}}^{t}_{log(ridge)}$ converges (i.e., when the discrete derivative $\Delta\hat{\boldsymbol{w}}_{log(ridge)} = \hat{\boldsymbol{w}}^{t}_{log(ridge)} - \hat{\boldsymbol{w}}^{t-1}_{log(ridge)} \approx \overrightarrow{\mathbf{0}}$).

Model validation on simulated data

We trained three models to get their predictions on the test set. The models were OLS regression, ridge regression with $\boldsymbol{w}_{TAL\ prior}$ (i.e., TAL-prior), and ridge regression with $\boldsymbol{w}_{TTB\ prior}$ (i.e., TTB-prior) for varying values of the θ penalty parameter (see below for the list of parameters). Then we computed the proportion of agreement between OLS regression and TAL-prior (Figure S1), as well as the the proportion of agreement between OLS regression and TTB-prior (Figure S2). This procedure was done for each of the 1000 iterations. As we can observe in Figures S1 and S2, both models have higher proportion of agreement with OLS for lower values of the θ penalty parameter and vice versa; lower agreement with OLS for high penalty values.

Before testing our no-covariance priors on real world data, we validated the model on 1000 iterations of simulated data, the same as in (Parpart, Jones & Love, 2018). We sampled twenty training observations ($n = 20$) for three cues ($m = 3$) — with 1 and 0 as possible values — from a uniform distribution. These vectors were then compared by their difference to obtain 190 possible pairwise difference vectors. The test set consisted of all possible difference vectors — with 1, -1, and 0 as possible values — but without the $\overrightarrow{\mathbf{0}}^T$ vector (i.e., 26 vectors total). The weight vector was created by sampling weights from an exponential distribution (rate parameter of 2). Thus to generate a binary valued $\boldsymbol{y}$, we multiplied the vertically concatenated training and test sets $\boldsymbol{X}$ with the weight vector $\boldsymbol{w}$ plus a column vector of i.i.d. (independently and identically distributed) Gaussian noise $\boldsymbol{\epsilon} = [\epsilon_1, ..., \epsilon_n]^T$ from a standard normal distribution and took the sign:

$$\boldsymbol{y} = \text{sign}(\boldsymbol{X}\boldsymbol{w} + \boldsymbol{\epsilon}) \tag{13}$$

$$\text{where } y_i \in \{-1, 1\}, \text{ since } p(y_i = 0) = 0$$

List of θ parameters

Applications I-II

0.0e+00, 1.0e-05, 2.5e-02, 5.0e-02, 7.5e-02, 1.0e-01, 2.0e-01, 4.0e-01, 6.0e-01, 8.0e-01, 1.0e+00, 2.0e+00, 4.0e+00, 6.0e+00, 8.0e+00, 1.0e+01, 2.0e+01, 4.0e+01, 6.0e+01, 8.0e+01, 1.0e+02, 2.0e+02, 4.0e+02, 6.0e+02, 8.0e+02, 1.0e+03, 2.0e+03, 9.5e+03, 4.5e+04, 2.1e+05, 1.0e+06, 2.0e+06, 5.3e+07, 1.4e+09, 3.8e+10, 1.0e+12, 1.0e+15, 1.0e+18, and 1.0e+21.

Application III

0.0e+00, 1.1e+00, 2.2e+00, 3.3e+00, 4.4e+00, 5.6e+00, 6.7e+00, 7.8e+00, 8.9e+00, 1.0e+01, 1.0e+02, 1.2e+03, 2.3e+03, 3.4e+03, 4.5e+03, 5.6e+03, 6.7e+03, 7.8e+03, 8.9e+03, and 1.0e+04.

Number of cues per dataset

Professors' Salaries: 4, Fish Fertility: 3, Fuel Consumption: 6, Attractiveness Men: 3, Land Rent: 4, High School Dropouts: 18, Attractiveness Women: 3, Cloud Rainfall: 6, Car Accidents: 13, Mortality Rates: 15, Body Fat: 14, Homelessness: 6, Oxygen: 5, Ozone in S.F.: 4, Mammals' Sleep: 9, Obesity: 10, Biodiversity: 6, Oxidants in L.A.: 4, City Size: 9, House Prices: 10, and Breast Cancer (Wisconsin): 9.

Splitting training data

In an alternative training scheme, we split the training data from the 20 classic datasets in half. From the 50 samples, 25 of them were used to learn the prior and the other 25 samples were used to learn the final regression weights along with the just learnt prior. This scheme not only helps avoid double counting of the data but it also permits learning an OLS prior too. This alternative training scheme is presented in Figure S7. The figure clearly shows the OLS-prior model performs worse on the majority of the datasets compared to the other three models. A

fair comparison with TAL-prior ($t(19) = 3.04$, $p = 0.007$) and the TTB-prior ($t(19) = 3.88$, $p = 0.001$) models confirms this.

Model variance

Furthermore, we can observe that the accuracy (i.e., generalisation performance) of the OLS-prior model presents more variance (measured in standard deviations) when compared to its counterparts in Figure S8. We confirm this observation with paired t-tests of the standard deviations of model accuracy (TAL-prior: $t(19) = 4.02$, TTB-prior: $t(19) = 4.14$, and Zero-prior models: $t(19) = 5.06$, all at $p < 0.001$).

Figures S1-S8

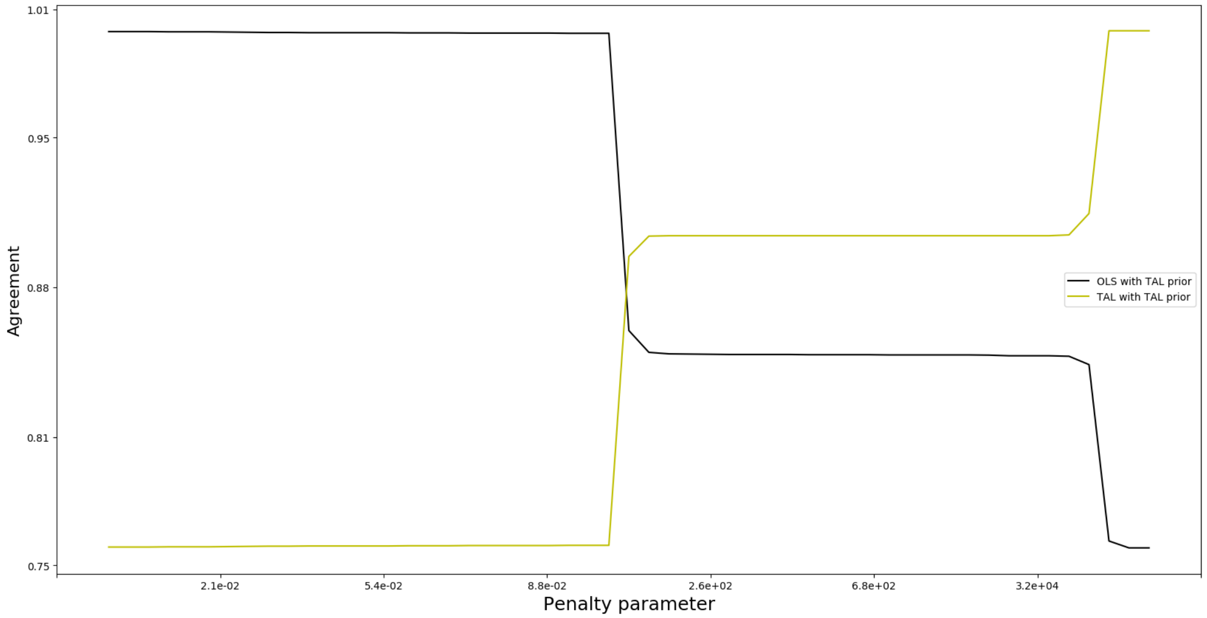

Figure S1: Proportion of agreement between test set predictions of OLS regression and TAL-prior (black) and the TAL heuristic with the TAL-prior model (yellow). The agreement was computed as the mean over 1000 iterations of simulated for varying degrees of the θ penalization parameter in the TAL-prior model.

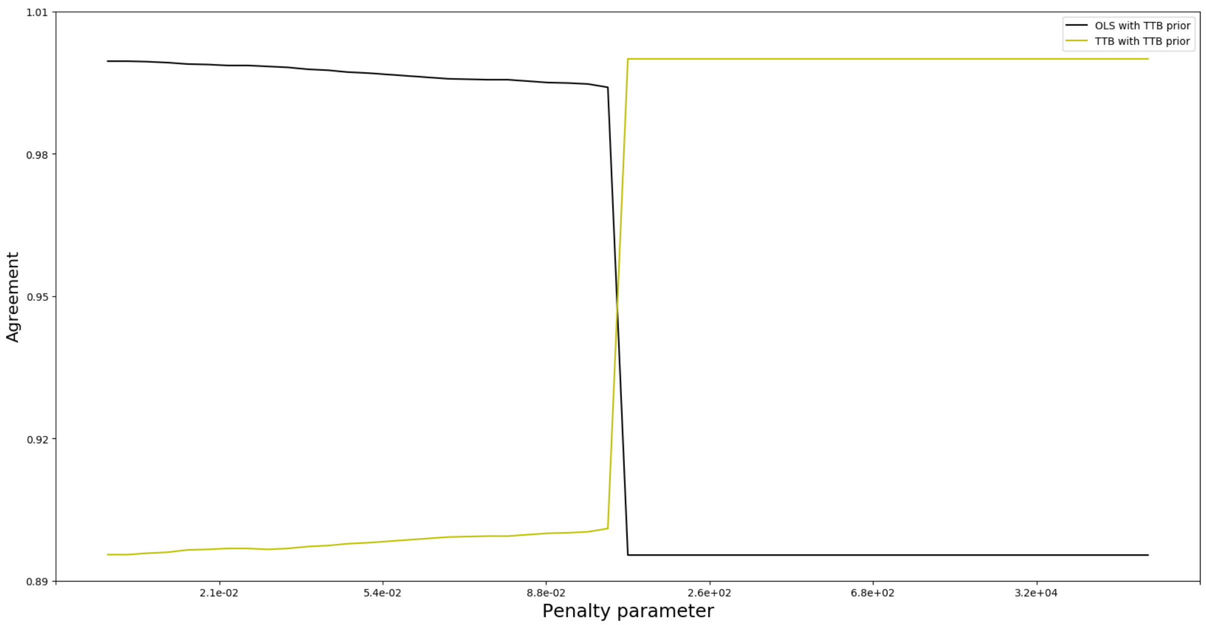

Figure S2: Proportion of agreement between test set predictions of OLS regression and TTB-prior (black) and the TTB heuristic with the TTB-prior model (yellow). The agreement was computed as the mean over 1000 iterations of simulated for varying degrees of the θ penalization parameter in the TTB-prior model.

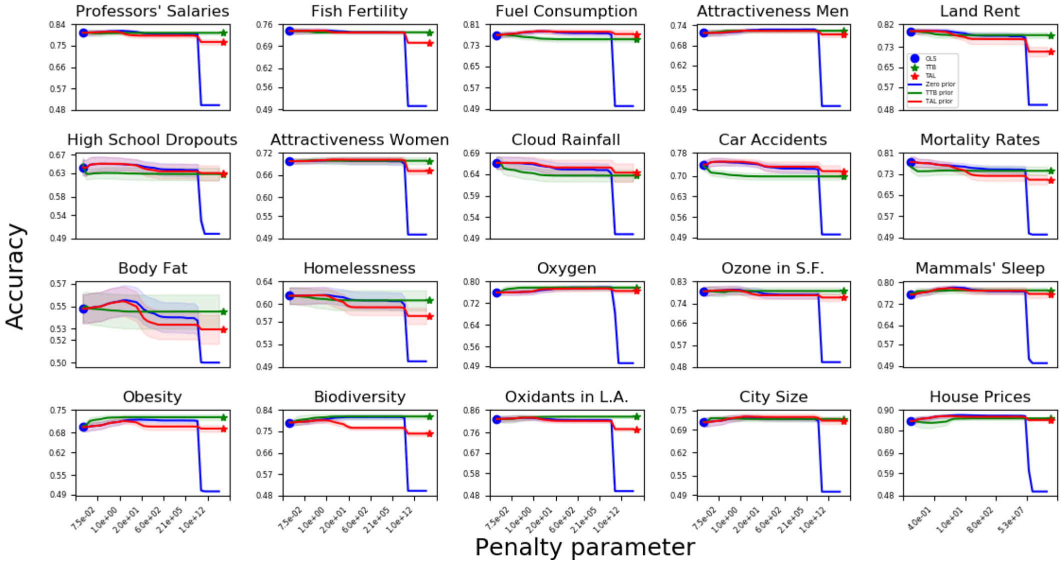

Figure S3: Generalization performance for 20 datasets (trained on 50 samples). The ordinate axis shows mean test accuracy for 1000 iterations of sampling observations for the training set. Zero prior refers to ridge regression with no prior on the regression weights (i.e., the prior is the zero vector). TAL-prior is ridge regression with $\boldsymbol{w}_{TAL\ prior}$ and TTB-prior is ridge regression with $\boldsymbol{w}_{TTB\ prior}$. TTB-prior may differ from OLS when $\theta = 0$ due to the modified design matrix $\boldsymbol{X}_{TTB}$ and differences in convergence of the Newton-Raphson algorithm. Shaded areas represent 1 standard deviation around each point.

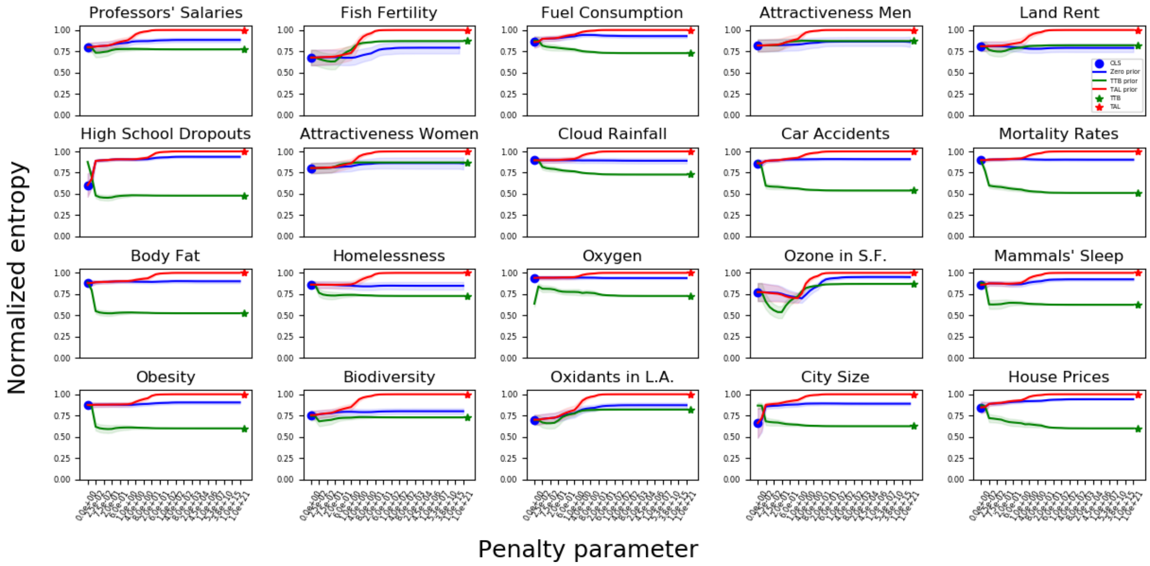

Figure S4: Normalized entropy for 20 datasets (trained on 50 samples). The ordinate axis shows mean normalized entropy for 1000 iterations of sampling observations for the training set. The same models are considered here as in Figure S3. Also, as in Figure S3, TTB-prior may differ from OLS when $\theta = 0$. Shaded areas represent 1 standard deviation around each point.

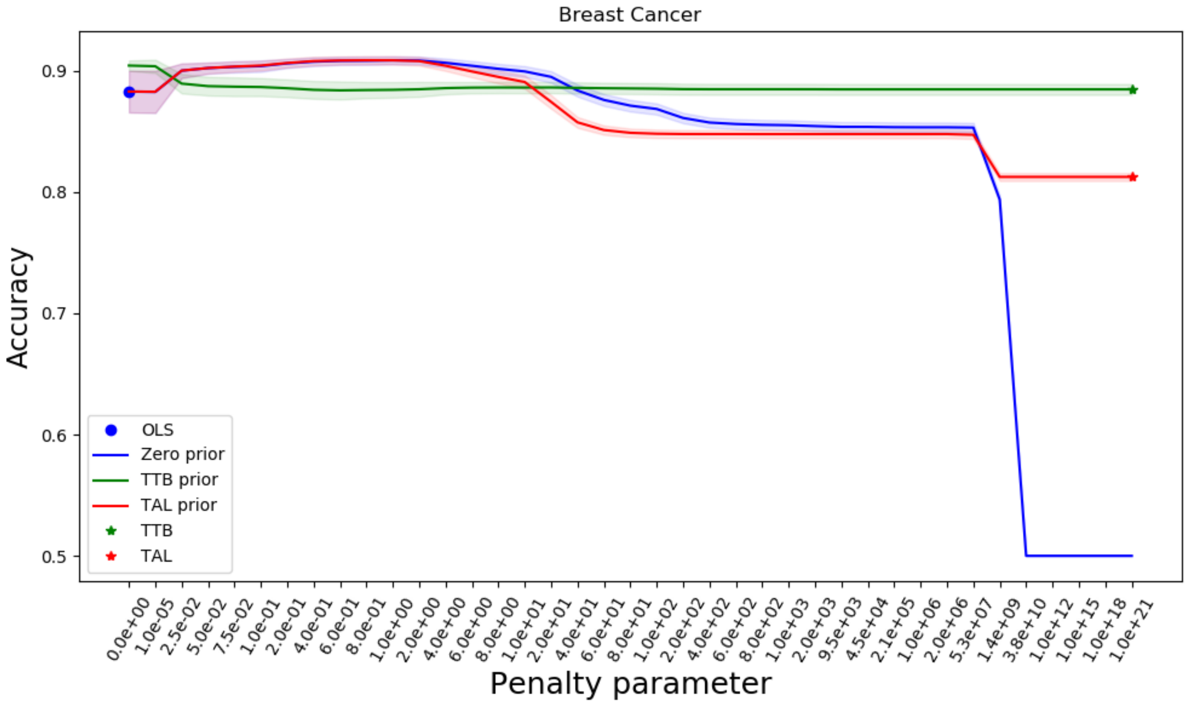

Figure S5: Generalization performance for Breast Cancer Wisconsin (Diagnostic) Data Set (trained on 100 samples).The ordinate axis shows mean test accuracy for 1000 iterations of sampling observations for the training set. The same models are considered here as in Application I (see Figure S3). Also, as in Figure S3, TTB-prior may differ from OLS when $\theta = 0$. Shaded areas represent 0.5 standard deviations around each point.

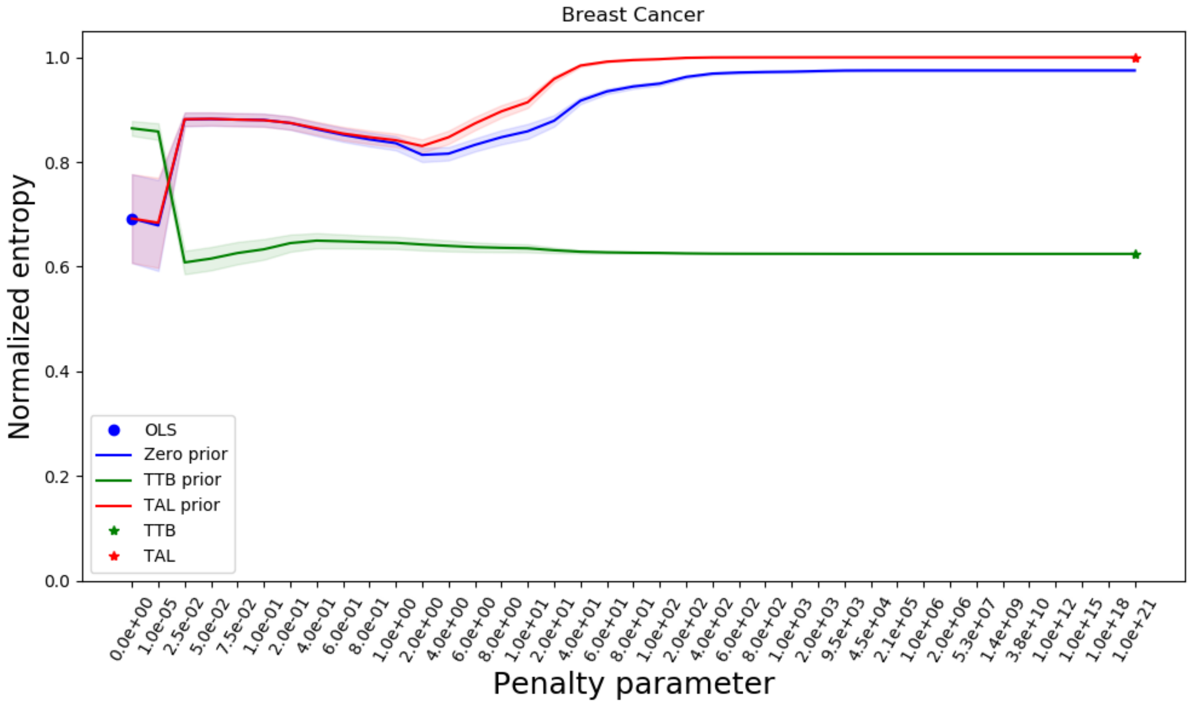

Figure S6: Normalized entropy for Breast Cancer Wisconsin (Diagnostic) Data Set (trained on 100 samples).The ordinate axis shows mean normalized entropy for 1000 iterations of sampling observations for the training set. The same models are considered here as in Application I (see Figure S4). Also, as in Figure S3, TTB-prior may differ from OLS when $\theta = 0$. Shaded areas represent 0.5 standard deviations around each point.

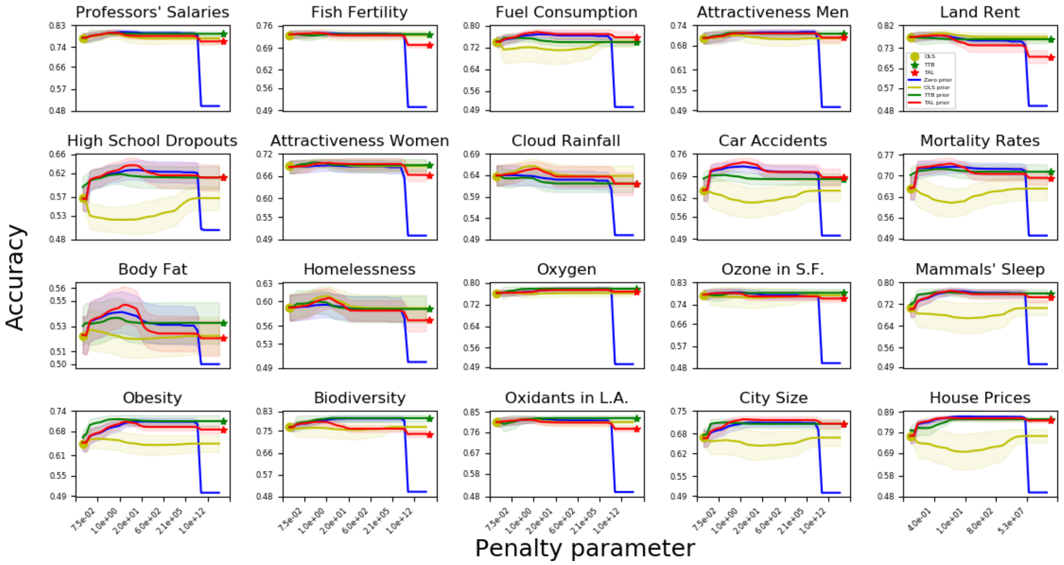

Figure S7: Generalization performance for 20 datasets with an alternative training scheme. Half the training samples (25) were used to learn the prior and the other half (25) were used to learn the regression weights. The ordinate axis shows mean test accuracy for 1000 iterations of sampling observations for the training set. Zero prior refers to ridge regression with no prior on the regression weights (i.e., the prior is the zero vector). OLS-prior is ridge regression with OLS weights as prior, TAL-prior is ridge regression with $\boldsymbol{w}_{TAL\ prior}$ and TTB-prior is ridge regression with $\boldsymbol{w}_{TTB\ prior}$. Shaded areas represent 1 standard deviation around each point.

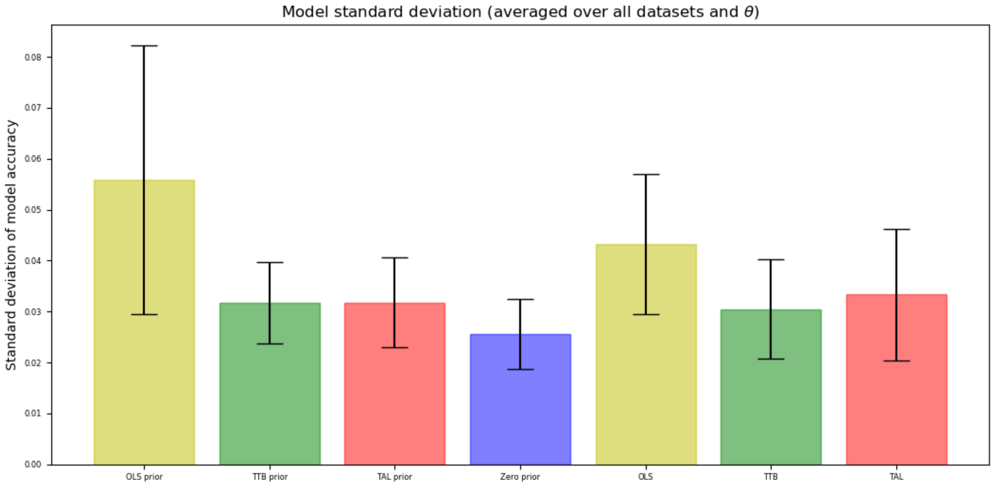

Figure S8: Standard deviation of the accuracy with an alternative training scheme. As in Figure S7, half the training samples (25) were used to learn the prior and the other half (25) were used to learn the regression weights. The ordinate axis shows the standard deviation of the mean test accuracy averaged over all hyperparameters θ and all 20 datasets. Zero prior refers to ridge regression with no prior on the regression weights (i.e., the prior is the zero vector). OLS-prior is ridge regression with OLS weights, TAL-prior is ridge regression with $\boldsymbol{w}_{TAL\ prior}$ and TTB-prior is ridge regression with $\boldsymbol{w}_{TTB\ prior}$.
